# Ability of the Analgesia Nociception Index variations to identify a response to a volume expansion of 250 mL of crystalloids in the operating room (REVANI): a prospective observational study

**DOI:** 10.1186/s12871-023-02181-2

**Published:** 2023-06-21

**Authors:** Hugues de Courson, Grégoire Chadefaux, Benjamin Abel, Delphine Georges, Eric Verchere, Matthieu Biais

**Affiliations:** 1 Centre Hospitalier Universitaire de Bordeaux, Bordeaux, France; 2 Service d’Anesthésie-Réanimation, Groupe Hospitalier Pellegrin, Place Amélie Raba Léon, 33000 Bordeaux, France

**Keywords:** Analgesia nociception index, Cardiac output, Non-invasive, Operating room, Volume expansion

## Abstract

**Background:**

Analgesia Nociception Index (ANI) is a device based on analysis of the R-R interval and respiratory sinus arrhythmia to assess the balance between sympathetic and parasympathetic activity. The autonomic system is directly affected by load changes. Therefore, monitoring sympathetic tone and its change could theoretically allow tracking of load changes during volume expansion. The aim of the present study was to determine whether changes in ANI are able to track the increase in stroke volume caused by volume expansion (SV).

**Methods:**

This prospective observational study included mechanically ventilated patients undergoing neurosurgery and benefiting from SV monitoring. Exclusion criteria were cardiac dysfunction, arrhythmias, beta-blockade therapy, and dysautonomia. SV was optimized by fluid administration of 250 ml of crystalloid fluid. A positive fluid increase was defined as a SV increase of 10% or more from baseline. Changes in SV and medium ANI (ANIm) were recorded before and 4 to 5 min after volume expansion.

**Results:**

Sixty-nine patients had 104 fluid challenges (36 positive and 68 negative). Volume expansion resulted in a greater ANI increase in responders than in nonresponders. The change in ANIm > 5 predicted fluid responsiveness with a sensitivity of 68.4% (95% CI: 67.4% to 69.5%) and a specificity of 51.2% (95% CI: 50.1% to 52.3%). The area under the receiver operating characteristic curve was 0.546 (95% CI: 0.544 to 0.549) and appeared to be affected by remifentanil dose and baseline ANI.

**Conclusion:**

Changes in ANIm induced by fluid challenge **is** not able to predict fluid responsiveness in mechanically ventilated patients undergoing neurosurgery.

**Trial registration:**

Clinical trial registration: NCT04223414.

## Background

Perioperative hemodynamic management aims to optimize stroke volume (SV) to achieve the best possible tissue oxygenation. Although hemodynamic optimization reduces postoperative morbidity and mortality, fluid overload can lead to pulmonary or peripheral edema and its associated side effects [1, 2]. Therefore challenge for physicians, is to accurately identify which patients will respond to fluid administration by increasing SV. Prediction of fluid responsiveness using dynamic indices based on heart–lung interactions during mechanical ventilation is nowadays severely limited by the widespread use of low-tidal ventilation strategies in the operating room [3]. Therefore, fluid titration by volume expansion and monitoring of its effects on SV is the most appropriate. This strategy is currently recommended by the French Society of Anesthesiologists [4] and the National Institute for Clinical Excellence [5]. Regardless of the approach, hemodynamic optimization needs to monitor SV which requires the use of invasive or semi-invasive [6] devices that limit their spread in daily practice. This emphasizes the need for the development of less invasive tools.

Heart rate variability analysis, based on electrocardiogram analysis and, in particular, on RR interval variations during the respiratory cycle, is now recognized as a simple, reliable, and non-invasive tool to assess the balance between sympathetic and parasympathetic activity [7–9]. MetroDoloris® has developed the Analgesia Nociception Index (ANI) based on RR analysis. The value of ANI can range from 0 to 100. A value of ANI close to 100 corresponds to a predominant parasympathetic tone (low-stress level, analgesia) and a value close to 0 corresponds to a predominant sympathetic tone (high-stress level, nociception). It is mainly used to monitor analgesia [10–14], but it has also been used for hemodynamics in some studies, for example, as a predictor of arterial hypotension after spinal anesthesia [15–18]. Indeed, sympathetic tone is directly affected by volemia, with the sympathetic nervous system being more stimulated and the parasympathetic nervous system withdrawing in hypovolemia [19]. Thus, the autonomic system is directly affected by load changes. Monitoring sympathetic tone and its variaitons could theoretically allow us to track the change in load during volume expansion. We hypothesized that the increase in SV induced by volume expansion would lead to a significant change in the orthosympathetic-parasympathetic balance in favor of parasympathetic tone and thus to an increased ANI value. This increase would be more pronounced in responders to fluid administration (significant increase in SV) than in non-responders. Therefore, volume expansion induced changes in ANI could detect volume expansion induced changes in SV.

The aim of the present study was to determine whether changes in ANI could detect fluid responsiveness following a volume expansion of 250 ml of crystalloids in the operating room.

## Methods

### Ethics approval

The study was performed in accordance with the Declaration of Helsinki. Ethical approval for this study (Ethics Committee N° ID-RCB: 2019-A01949-48) was obtained from the Comité de Protection des Personnes Ile de France X, France, on November 14, 2019 (Pr P. Casassus). In accordance with French law, all patients were provided with written information about the study and their informed consent to participate was obtained.This study has been registered on Clinicaltrials.gov: NCT04223414.

This manuscript complies with the applicable guidelines of STROBE.

### Patients

This was a prospective, observational study conducted in a tertiary University Hospital from January 2020 to June 2020. Inclusion criteria were: patients older than 18 years old who were scheduled for neurosurgery, equipped with a radial arterial catheter and cardiac output monitor. Exclusion criteria included emergency surgery, cardiac dysfunction, arrhythmia, beta-blockade therapy use, pacemaker, intracranial hypertension, pregnancy, dysautonomia and refusal to participate.

### Anesthesia protocol

All patients received of total intravenous anesthesia achieved by target-controlled infusion of propofol and remifentanil. Administration of a non-depolarizing neuromuscular blocker (atracurium) was at the discretion of the attending physician. Patients received volume-control mechanical ventilation (tidal volume of 6–8 ml.kg^−1^ of ideal body weight; positive end-expiratory pressure was set at 6 to 8 cmH_2_O.

### Hemodynamic monitoring

Before induction of anesthesia, the patient was monitored by non-invasive blood pressure measurement, pulse oximetry and ECG. Immediately after induction of anesthesia, a radial artery catheter was inserted and connected to the bedside monitor for invasive arterial pressure monitoring and to a dedicated transducer (ProAQT™, Maquet, Rastatt, Germany) for monitoring SV, cardiac output, pulse pressure variations (PPV), and stroke volume variations (SVV).

### ANI monitor

ANI monitor v1 (Metrodoloris™, Lille, France) was directly connected to an ECG monitor and allowed analysis of heart rate. ANI is expressed as two indices between 0 and 100: the mean ANI (ANIm) is the value of ANI averaged over the last 4 min and the instantaneous ANI (ANIi) is averaged over a shorter period of 80 s, with each elementary measurement taken over 64 s. A ANI value close to 100 corresponds to predominantly parasympathetic tone, whereas a value close to 0 corresponds to predominantly sympathetic tone. The physician in charge of anesthesia was not aware of the value of ANI and did not use it to perform the anesthesia.

### Study design

Fluid challenge was performed at the discretion of the physician according to standard recommendations [4, 5] and consisted of infusion of 250 ml of crystalloid (0.9% saline) over 10 min. Data were collected immediately before and 4–5 min after fluid infusion. Bolus administration or changed in vasopressor dosage, as well as changes in ventilatory parameters or anesthesia drugs dosage, were not allowed during fluid infusion. Multiple fluid challenges could be performed in the same patient depending on whether SV had been previously increased by more than 10% or at the discretion of the physician according to recommendations.

### Statistical analysis

Response to volume expansion was defined as an increase in SV of 10% or more. We hypothesized a proportion of 30% of responders to volume expansion [20]. To detect an area under the receiver operating characteristic curve (AUROC) ≥ 0.76 with a power of 90% and an alpha value of 0.05, a minimum number of 56 subjects was required [21]. Because several parameters could lead to subsequent exclusion (changes in propofol or remifentanil dosage, bolus of vasopressor, changes in ventilatory setting during the study protocol), we planned to include at least 10 more patients (minimum of 66 patients).

Quantitative variables were described by mean (standard deviation) or median [interval inter-quartile] according to their distribution. Qualitative data were described by their number (percentage). Data collected before and after volume expansion were compared with a paired Student t test or a paired Mann–Whitney test, depending on their distribution. The relationship between ANIm and SV variation was assessed by using a linear correlation. Diagnostic performance of change in ANI to diagnose fluid responsiveness was estimated using Receiver operating characteristic curves, the AUROC, and their confidence interval (CI). Because of the repeated fluid challenges, CI were estimated by using an individual Boostrap method with 1000 replication. The best cut-off value was by maximizing the Younden index (sensitivity + specificity – 1). We tested the diagnostic performance of changes in ANI during volume expansion according to remifentanil dose regimen and baseline ANI value using the Faraggi’s method [22]. A *p*-value less than 0.05 was considered significant. All analyzes were performed using R Development Core Team (http://www.R-project.org), version R 4.0., accessed June 2020).

## Results

### Patients

**Fig. 1:**
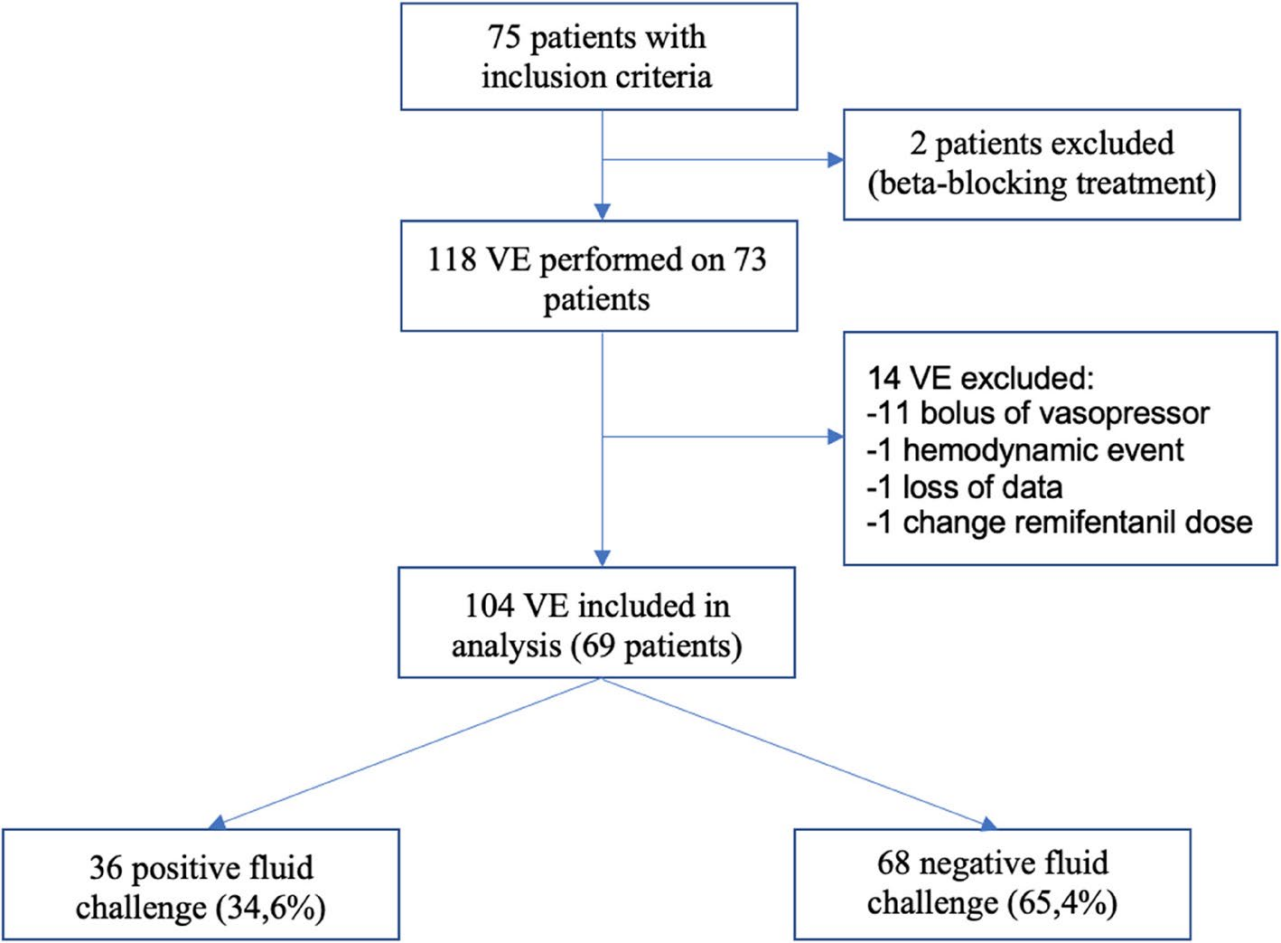
Flow chart (VE: Volume expansion)

Of the 75 patients included, 2 were excluded because of beta-blocking medication use (Fig. 1). 118 volume expansions were performed, of which 14 were excluded mainly because of the use of bolus vasopressors during volume expansion. A total of 104 volume expansions (36 positives and 68 negatives) fluid challenges were performed in 69 patients. Table 1 provides an overview of the patients’ characteristics.

**Table 1:** Characteristics of patients included in analysis (*n* = 69)

**Characteristics**	
**Age, yr**	57 (14)
**Sex, Female, n (%)**	35 (51)
**ASA physical status (%)**	
1	1 (2)
2	21 (30)
3	47 (68)
**BMI, kg.m** ^**−2**^	26 (5)
**Body weight, kg**	72 (16)
**Ideal body weight, kg**	62 (7)
**Comorbidities**	
Chronic hypertension	11 (16)
Diabetes	1 (1)
Tabacco	21 (30)
Alcohol consumption	5 (7)
Stable cardiovascular disease	3 (4)
Stable pulmonary disease	13 (19)
**Treatment, n (%)**	
Antihypertensive	11 (16)
Including CEI/ARBs	10 (15)
**Position, n (%)**	
Supine position	46 (67)
Partial lateral decubitus	23 (33)
**Surgery, n (%)**	
Brain tumor	58 (84)
Aneurysm	6 (9)
Others	5 (7)
**Length of surgery, h**	3 (1)
**Tidal volume of ideal body weight (ml.kg** ^**−1**^ **)**	7.2 (0.7)
**Ventilatory frequency (cycles.min** ^**−1**^ **)**	14 [12 – 1]
**PEEP (cmH**_**2**_ **0)**	6 [5, 6]
**Number of patients receiving**	
1 fluid challenge, n	41
2 fluid challenges, n	19
3 fluid challenges, n	6
4 fluid challenges, n	3

Values are mean (standard deviation), median [25–75% interquartile range] or number (%) as appropriate

*ACEi* Angiotension Conversion Enzyme inhibitor, *ARBs* Angiotensin Receptor Blockers

T0: baseline

T1: 4–5 min after the end of volume expansion

### Effects of volume expansion

Table 2 summarizes the hemodynamic, ventilatory, and ANI variables according to positive or negative fluid challenges. Heart rate, Pulse Pressure Variation, and Stroke Volume Variation decreased significantly after the fluid challenge in both groups. Stroke volume and ANIm increased during positive and negative fluid challenge. Volume expansion resulted in a 15.8(5.3)% increase in cardiac output in positive fluid challenges and a 1.0(6.8)% increase with negative fluid challenges.

**Table 2:** Haemodynamic and ANI variables before and after fluid challenge in positive (*n* = 36) and negative fluid challenge (*n* = 68)

	**Before volume expansion**		**After volume expansion**		***p*** **-value**
**Heart rate (beat.min** ^**−1**^ **)**					
Negative fluid challenges	72	[64 – 81]	71	[60 – 79]	< 0.001
Positive fluid challenges	67	[62 – 73]	62	[58 – 70]	< 0.001
**Mean arterial pressure (mmHg)**					
Negative fluid challenges	69	[57 – 77]	67	[57 – 78]	0.807
Positive fluid challenges	67	[60 – 71]	68	[61 – 73]	0.210
**Stroke volume (ml)**					
Negative fluid challenges	38	[34 – 53]	40	[36 – 55]	< 0.001
Positive fluid challenges	37	[33 – 43]	43	[38 – 48]	< 0.001
**Cardiac output (l.min** ^**−1**^ **)**					
Negative fluid challenges	2.8	[2.3 – 3.7]	2.9	[2.3 – 3.8]	0.222
Positive fluid challenges	2.5	[2.0 – 3.1]	2.9	[2.3 – 3.3]	< 0.001
**PPV (%)**					
Negative fluid challenges	12	[8–15]	9	[7–13]	< 0.001
Positive fluid challenges	11	[9–17]	8	[6–11]	< 0.001
**SVV (%)**					
Negative fluid challenges	14	[11–20]	12	[9–16]	< 0.001
Positive fluid challenges	14	[11–17]	9	[8–12]	< 0.001
**Remifentanil dose (ng.ml** ^**−1**^ **)**					
Negative fluid challenges	4.0	[3.0 – 4.6]	4.0	[3.0 – 4.6]	-
Positive fluid challenges	3.5	[2.7 – 4.0]	3.5	[2.7 – 4.0]	-
**Propofol dose (μg.ml** ^**−1**^ **)**					
Negative fluid challenges	4.0	[3.8 – 5.0]	4.0	[3.8 – 5.0]	-
Positive fluid challenges	3.7	[3.1 – 4.0]	3.7	[3.1 – 4.0	
**ANI mean (n)**					
Negative fluid challenges	70	[54 – 82]	77	[58 – 86]	0.019
Positive fluid challenges	69	[62 – 84]	79	[66 – 91]	0.005

Positive fluid challenge defined as a stroke volume increase ≥ 10%. Values are median (25^th^ to 75^th^ percentile)

*ANI* Analgesia Nociceptive Index, *PPV* Pulse pressure variations, *SVV* Stroke volume variations

### Diagnosis performance of fluid responsiveness

**Fig. 2:**
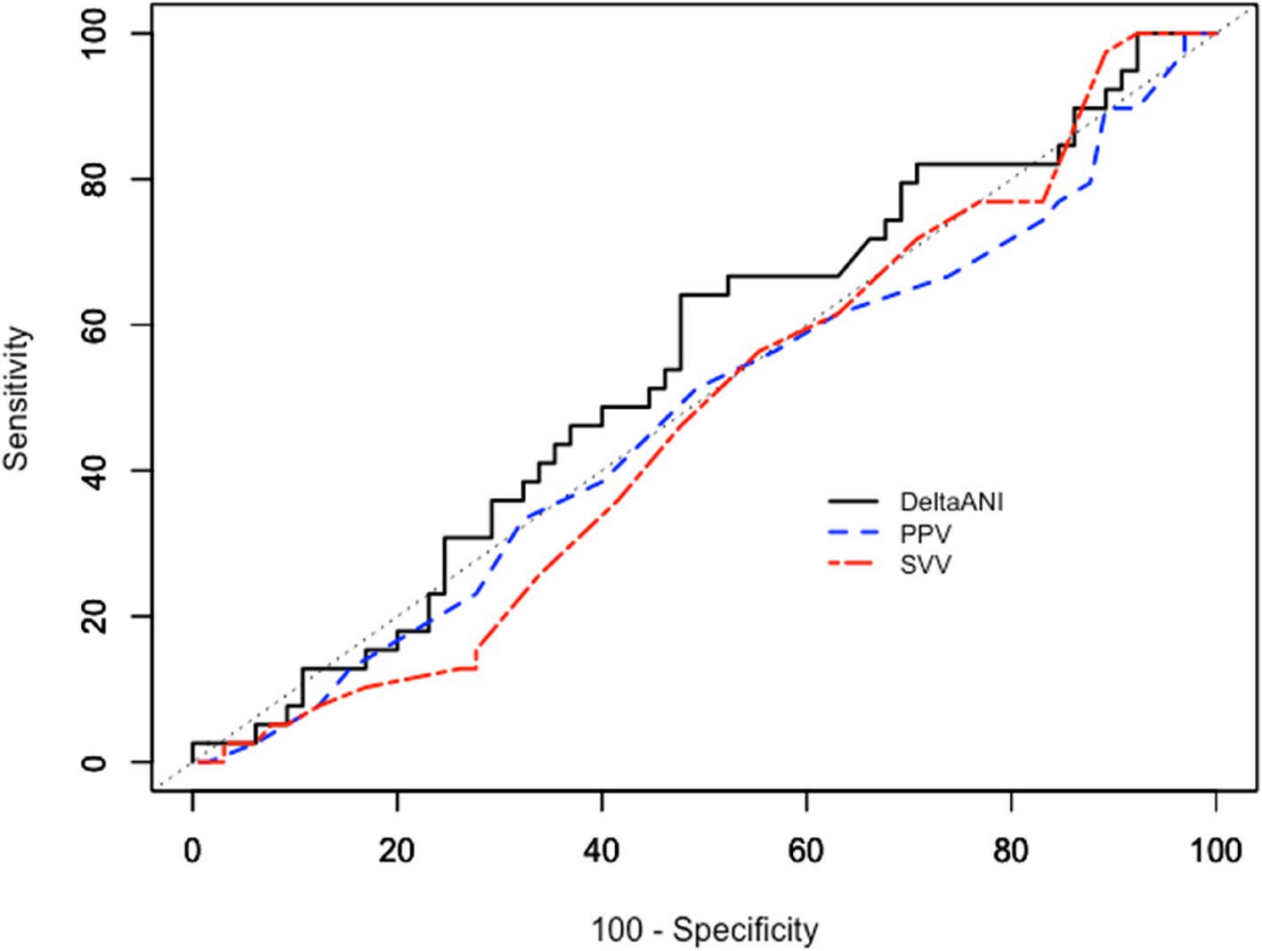
Receiver operating characteristics (ROC) curves evaluating the ability of baseline PPV and SVV, and of changes in Analgesia Nociceptive Index (ANI) to detect an increase in stroke volume ≥ 10% following a volume expansion of 250 mL saline 0.9%

The diagnostic performance of PPV, SVV and changes in ANI for predicting fluid responsiveness are shown in Table 3. Positive predictive value was 0.468 (0.464–0.473) and negative predictive value was 0.751 (0.746–0.757). AUCROC for changes in ANIm to detect a stroke volume increase of 10% or more after volume expansion was 0.546 (95% CI, 0.544 to 0.549) (Fig. 2). The best threshold was 5%, corresponding to a sensitivity of 68.4% (95% CI, 67.4 to 69.5) and a specificity of 51.2%. (95% CI, 50.1 to 52.3).

**Table 3:** Diagnostic Performance of PPV, SVV and changes in ANIm

	**AUC**	**Best threshold**	**Specificity**	**Sensitivity**	**PV + **	**PV-**	**LR + **	**LR-**	**Youden**	**P value**
Diagnosis performance for detecting fluid responsiveness defined by an increase in SV more than 10%										
DeltaANIm	0,479 [0,340 – 0,617]	11%	0,568 [0,405 – 0,730]	0,500 [0,312 – 0,656]	0,500 [0,367 – 0,625]	0,568 [0,455 – 0,677]	1,156 [0,634 – 1,854]	0,881 [0,535 – 1,374]	0,068	ref
SVV	0,464 [0,326 – 0,603]	7%	0,054 [0,000 – 0,135]	1,000 [0, 0, 1]	0,478 [0,464 – 0,500]	1,000 [0, 0, 1]	1,057 [0,979 – 1,142]	0,000 [0–6, 86]	0,054	0,887
PPV	0,525 [0,385 – 0,664]	19%	0,865 [0,757 – 0,973]	0,250 [0,094 – 0,406]	0,625 [0,357 – 0,867]	0,571 [0,517 – 0,632]	1,850 [0,666 – 8,252]	0,867 [0,657 – 1,076]	0,115	0,634
Diagnosis performance for detecting fluid responsiveness defined by an increase in CO more or equal than 10%										
DeltaANIm	0,614 [0,464 – 0,763]	12%	0,490 [0,347 -,0633]	0,750 [0,550 – 0,900]	0,375 [0,286 – 0,471]	0,829 [0,706- 0,935]	1,470 [0,975 – 2,129]	0,510 [0,152 – 1,019]	0,828	ref
SVV	0,546 [0,399 – 0,694]	14%	0,633 [0,490 – 0,755]	0,550 [0,350 – 0,750]	0,379 [0,250 – 0,522]	0,775 [0,684 – 0,872]	1,497 [0,804 – 2,538]	0,711 [0,352 – 1,122]	0,183	0543
PPV	0,555 [0,406 – 0,704]	12%	0,633 [0,510 – 0,755]	0,600 [0,400 – 0,800]	0,400 [0,276 – 0,533]	0,795 [0,702 – 0,895]	1,633 [0,933 – 2,768]	0,632 [0,290 – 1,036]	0,795	0,575

*ANI* Analgesia Nociceptive Index, *AUC* Area Under Curve, *CI* Confidence Interval, *ΔANIm* Changes in ANI following volume expansion, *LR-* Negative Likelihood Ratio, *LR + * Positive Likelihood Ratio, *PV + * Positive Predictive Value, *PV-* Negative Predictive Value, *PPV* Pulse Pressure Variations, *SV* Stroke Volume, *SVV* Stroke Volume Variations

**Fig. 3:**
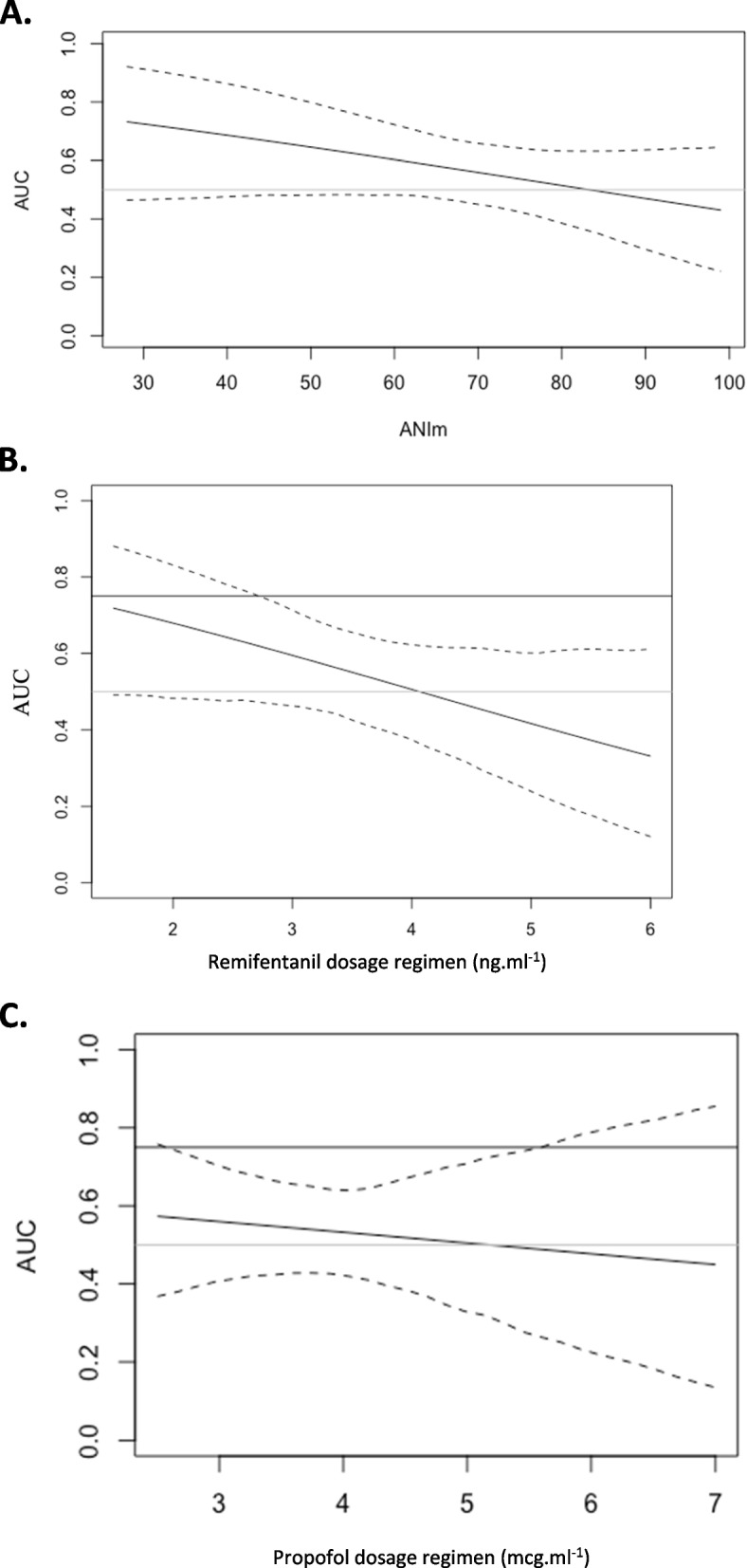
Conditional values of the area under the receiver operating characteristic curve (AUC) generated to test the ability changes in Analgesia Nociceptive Index (ANI) to detect an increase in stroke volume ≥ 10% following a volume expansion of 250 mL saline 0.9% according to. **a** Mean Analgesia Nociceptive Index (ANIm) before volume expansion. **b** Remifentanil dose regimen (ng.ml^−1^). **c** Propofol dose regimen (mcg.ml^−1^). Continuous lines show the mean area under the receiver operating characteristics curve and dotted lines show the 95% confidence interval. The area under the curve, which reflects diagnostic ability, appears to be influenced by the dose of remifentanil and the ANI value. High remifentanil dose and high ANIm value seem to be associated with lower diagnostic abilities

Diagnostic performances of changes in ANI to predict fluid responsiveness appeared to differ according to baseline levels of ANI and the remifentanil dosing regimen but did not appear to be affected by the propofol dosing regimen (Fig. 3). The ability of changes in ANI to detect fluid responsiveness tended to improve as remifentanil concentration decreased. Similarly, high baseline levels of ANI appeared to alter the diagnostic performance of changes in ANI.

## Discussion

This study suggests that (i) volume expansion induces a significant increase in ANIm values that is more pronounced in positive fluid challenge than in negative fluid challenges and that (ii) ANIm variations after a volume expansion of 250 ml of crystalloids are not able to diagnose fluid responsiveness in neurosurgical patients ventilated with low tidal volume in the operating room. Diagnostic performance of ANIm variations appears to be affected by remifentanil dose.

Even though the beneficial effect of perioperative hemodynamic optimization has been demonstrated, it is rarely performed in daily practice nowadays because of the need for devices to monitor stroke volume. The whole challenge is to develop easy-to-use and non-invasive monitoring. Dynamic parameters seemed promising but as confirmed in our study, fail to properly diagnose fluid responsiveness. Recently, the use of End-Tidal CO_2_ variations following volume expansion in mechanically ventilated patients was also investigated, but they were also unable to diagnose fluid responsiveness [23].

### Volume expansion induced changes in ANI

The occurrence of relative or absolute hypovolemia results in a decrease in stroke volume due to a decrease in systemic venous return. In response, orthosympathetic stimulation and reversal of parasympathetic braking occur. In physiological situations, reversal of this balance leads to cardiovascular reactivity (increased heart rate, arterial and venous vasoconstriction) and activation of the renin-angiotensin system [24]. Thus, performing a volume expansion in a hypovolemic situation results in a reversal of the sympathovagal balance.

As expected, ANIm increased with both positive and negative fluid challenges, but increased more so in positive fluid challenges.

According to previous studies, heart rate variability (HRV) analysis shows promise. For example, in 2010, Ryan et al*.* studied HRV as a marker of hypovolemia in 101 healthy volunteers who were experimentally immersed in a hypovolemic situation using the lower body negative pressure technique [25]. The authors found a strong correlation between changes in HRV markers and changes in cardiac output, although they did not find a correlation between changes in HRV and the depth of hypovolemia. These findings were replicated by the work of Lin's team, which examined the effects of changes in blood volume induced by standardized maneuvers in 26 healthy volunteers [26]. Performing of a "head-up tilt test" simulating hypovolemia resulted in a decrease in HRV, whereas a passive leg-raising maneuver induced an increase in HRV. In 2015, Elstad et al*.* replicated this experience in 10 healthy volunteers placed in hypovolemic situations after spontaneous and assisted ventilation [27]. During spontaneous ventilation, variations in HRV allowed detection of a hypovolemic situation (AUC = 0.81), and under assisted ventilation, experimental hypovolemic situations also induced changes in HRV (AUC = 0.76). In a different vein, Hanss [15, 16] and colleagues reported in the 2000s that HRV predicts severe hypotension after administration of regional anesthesia (spinal anesthesia for elective cesarean delivery) or general anesthesia [17]. They conclude that the HRV analysis would be useful in identifying patients at risk of severe arterial hypotension during spinal anesthesia. While these HRV analyzes require software for data acquisition and processing, ANI has been established in the field of anesthesia as an easy-to-use software initially developed to prevent hemodynamic changes after a nociceptive phenomenon. In 2016, Boselli et al*.* studied hemodynamic reactivity using the dynamic variations of ANI: a decrease of ANI of more or equal to 19% in 1 min has a high probability (AUROC = 0.90) to predict an increase of heart rate and/or systolic blood pressure of more than 20% within the next 5 min [28].

Our work is one of the first to address changes in ANI after a change in hemodynamics and differs from the previously cited studies in that it includes mechanically ventilated subjects and subjects under general anesthesia, which is more in line with reality than not. Indeed, the included population was similar in terms of fluid responsiveness to the work of Mac Donald et al*.*, who in their substudy of OPTIMISE Trial, reported only 29% of volume expansion that increased stroke volume [29] and Biais et al*.,* found 32% of positive fluid challenge in their study [20].

### Diagnosis performance of ANI

The poor diagnostic capabilities of ANI can be explained by two elements: small variations in cardiac output and general anesthesia. First, we observed a small increase in cardiac output, even in responder patients (16%). We can hypothesize that these fluctuations are not sufficient to produce a significant variation in ANI. Second, general anesthesia likely had a significant effect on our results. We have shown that remifentanil dose affects AUROC, which makes sense because the use of opioids during anesthesia serves in part to control the sympathetic response to a surgical stimulus. In our study, ANIm was 74 (59 – 86) before the initiation of the fluid challenge, likely indicating opioid overdose. The area under the ROC curve decreases with high baseline ANIm values and high remifentanil concentrations (Fig. 3). ANIm values did not affect the anesthetic protocol and were assessed independently during data analysis. Propofol may also affect this response, as has been shown in many studies demonstrating that propofol significantly decreases sympathetic nervous system (SNS) activity and its ability to respond to hypovolemia [30, 31]. In the present study, propofol did not appear to affect the diagnostic ability of ANI variations to identify fluid responsiveness.

### Stroke volume versus arterial pressure

As discussed above, most studies have focused on changes in blood pressure and/or heart rate induced by a nociceptive stimulus. However, few studies have focused on the analysis of cardiac output [25].

Blood pressure is a finely regulated variable and the adjustment of cardiovascular functions involves, including cardiac output, systemic vascular resistance, and blood volume. These regulatory mechanisms can act in the short term particularly with the help of the autonomic nervous system. A change in blood pressure leads to a response of the cardiovascular center with the help of the orthosympathetic and parasympathetic systems, which modulate cardiac output (especially via HR and myocardial contractility) but also systemic vascular resistance [32, 33].

Therefore, monitoring cardiac output seems even more important as it can be done by pulse wave analysis.

### Limitations

The present work has some limitations. First, this monocentric study, which included patients undergoing neurosurgery in the operating room did not include patients with arrhythmia or with a pacemaker, right or left heart failure or beta-blocker treatment limiting extrapolation of the results. Second, although ANI is well established in the field of anesthesia as an assessment of sympathovagal balance over the R-R interval, no other methods of HRV were analysis available to us. Thus, the calculation algorithms based on the amplitude measurement of the respiratory modulations of the RR series and the way in which the ANI value varies between 0 and 100 are not known precluding a thorough scientific analysis. One of the strengths of this study is the use of this new monitoring model in the field of anesthesia. In addition, some studies [34, 35] have found a tendency to increase the ratio between parasympathetic and sympathetic tone in right lateral decubitus. The partial lateral decubitus used in our study may have contributed to the lack of change in ANIm values following fluid challenge.

Third, cardiac output was monitored by using pulse contour analysis with a specific transducer (ProAQT®, Pulsion Medical System). Pulse contour analysis provides an estimate of SV by initial autocalibration without external calibration which may not be effective in the presence of vasoplegia or other changes in vascular resistance. However, Monnet et al. concluded that Pulsioflex is reliable for tracking fluid-induced changes in cardiac index [36] and recently, our team found a least significant changes of SV smaller than 10% threshold, making it possible to identify the effects of fluid administration [37].

Finally, the infusion regimen of remifentanil was left to the discretion of the attending physician and was not fixed. Large doses of remifentanil, by decreasing sympathetic response, may have altered the effects of volume expansion on ANI as shown in Fig. 3B.

## Conclusions

Our study suggests that ANI monitoring is not able to detect the hemodynamic response to volume expansion of 250 ml of crystalloid in mechanically ventilated patients undergoing general anesthesia, which appears to be influenced by the remifentanil dosing regimen and baseline values ANI.

## Data Availability

The datasets used and/or analysed during the current study are available from the corresponding author on reasonable request.

## Abbreviations

ANI: Analgesia Nociception Index
AUROC: Area Under the Receiver Operating Characteristic Curve
CI: Confidence interval
ANIm: Medium ANI
PPV: Pulse Pressure Variations
SV: Stroke Volume
SVV: Stroke Volume Variations
